# Metamizole versus ibuprofen at home after day surgery: study protocol for a randomised controlled trial

**DOI:** 10.1186/s13063-016-1586-8

**Published:** 2016-09-26

**Authors:** Björn Stessel, Michiel Boon, Elbert A. Joosten, Jean-Paul Ory, Stefan Evers, Sander M. J. van Kuijk, Jasperina Dubois, Daisy Hoofwijk, Luc Jamaer, Wolfgang F. F. A. Buhre

**Affiliations:** 1 Department of Anesthesiology and Pain Treatment, Jessa Hospital – Hasselt, Virga-Jesse Campus, Stadsomvaart 11, 3500 Hasselt, Belgium; 2 Department of Anesthesiology, Maastricht University Medical Center+, Maastricht, The Netherlands; 3 Department of Clinical Epidemiology and Medical Technology Assessment, Maastricht University Medical Center+, Maastricht, The Netherlands

**Keywords:** Ambulatory surgery, Day surgery, Metamizole, Dipyrone, NSAID, Recovery, Functional Recovery Index, EQ-5D, Acute pain, Postoperative pain

## Abstract

**Background:**

Postoperative pain and, in a more extended perspective, quality of recovery (QOR) should be considered the principal endpoints after day surgery. Non-steroidal anti-inflammatory drugs (NSAIDs) and paracetamol are a cornerstone of pain treatment after painful day surgery. Nevertheless, NSAIDs are not always sufficiently effective, have numerous contraindications, and consequently are not suitable in up to 25 % of all patients. Metamizole is a non-opioid compound with a favourable gastrointestinal, cardiovascular and cerebrovascular profile compared to NSAIDs. The aim of this study is to assess if a combination of metamizole and paracetamol is non-inferior to a combination of ibuprofen and paracetamol in the treatment of acute postoperative pain at home after painful day case surgery. In addition, we aim to assess and compare quality of recovery (QOR) profiles of both groups.

**Methods/Design:**

This is an investigator-initiated, double-blind, randomised controlled, non-inferiority trial. A total of 200 patients undergoing elective haemorrhoid surgery, arthroscopic shoulder or knee surgery, or inguinal hernia repair in a day care setting will be randomised to receive either a combination of metamizole and paracetamol (MP) or a combination of ibuprofen and paracetamol (IP). Participants will take study medication orally for 4 days. Primary endpoints are average postoperative pain intensity measured by an 11-point Numeric Rating Scale at postoperative day 1 and QOR profile measured by the Functional Recovery Index (FRI), the 1-item Global Surgical Recovery (GSR) index and the EuroQol (EQ-5D) questionnaire at days 1, 2, 3, 4, 7, 14 and 28 postoperatively. Secondary outcomes include compliance with study medication, adverse effects of study medication, use of rescue medication and satisfaction with study medication, surgery and hospital care and telephone follow-up.

**Discussion:**

This study will provide clinical evidence on the analgesic efficacy and safety of a combination of metamizole and paracetamol in treating postoperative pain at home after painful day surgery. This study may also provide an insight into QOR profile after four different types of surgery and into the interrelationship between three different instruments used to assess QOR.

**Trial status:**

Recruitment is currently ongoing.

**Trial registration:**

European Union Clinical Trials Register 2015-003987-35. Registered 10 November 2015.

## Background

Day surgery has been expanding substantially in the past decade, primarily because it is associated with lower costs and it is believed to be as safe as surgery in the inpatient setting. Moreover, it seems that early discharge can contribute to faster recovery and a decreased incidence of hospital-associated complications.

In view of the relative absence of major complications, postoperative pain and, in a more extended perspective, quality of recovery (QOR) should be considered the principal endpoints after day surgery [1–3]. Pain and QOR are two related phenomena encompassing many dimensions in physical, psychological and social health [4].

Particularly in the ambulatory setting, good postoperative analgesia is challenging because patients have to control pain at home by themselves [5] and the types of analgesics (i.e. no strong opioids) as well as the route of administration (i.e. no epidural, intravenous, subcutaneous or intramuscular route) is limited compared to the inpatient setting. Despite increased awareness and improvements in postoperative pain management over the last decades, the prevalence of outpatients suffering moderate to severe acute postoperative pain at home still remains high and varies from 9 to 40 % [6–10]. More specific, patients undergoing haemorrhoid surgery, arthroscopic shoulder and knee surgery, and inguinal hernia repair seem to be at highest risk to develop moderate to severe pain and to be poorly recovered on the fourth postoperative day [3, 8].

Another major disadvantage of the ambulatory setting is related to the absence of postoperative surveillance by professionals. The latter implicates that the individual patient has to assess, without any support, if his/her QOR is normal or not. Unfortunately, there is rather limited information on procedure-specific QOR after day surgery in a more protracted perspective [3, 4, 11]. It is of major importance to study QOR profiles after different types of day surgery. This knowledge would allow discriminating between a normal and pathological health trajectory. Furthermore, the role and effect of pain therapy on QOR profile should be more closely investigated.

Nowadays, a multimodal approach to control pain has been advocated in the ambulatory setting. This approach is based on a combination of paracetamol, non-steroidal anti-inflammatory drugs (NSAIDs), weak opioids, and local and regional anaesthesia has been advocated in the ambulatory setting [7, 12, 13]. Furthermore a systematic meta-analysis has shown that a combination of paracetamol and an NSAID may offer superior analgesia compared to either drug alone [14]. Consequently, ibuprofen, a non-steroidal anti-inflammatory drug (NSAID) with a favourable analgesic profile [15] in combination with paracetamol comprise our standard multimodal pain treatment model for patients at home after painful day surgery. Nevertheless, NSAIDs are not always sufficiently effective [8], have numerous contraindications [16–20], and as a result of this are not suitable in up to 25 % of all patients [21].

Metamizole or dipyrone is a non-opioid compound with potent analgesic, antipyretic and spasmolytic effects [22].

The analgesic efficacy of intravenous or intramuscular metamizole for pain relief after inpatient surgery is well described [23–29]. The analgesic efficacy of individual tramadol, metamizole and paracetamol for postoperative analgesia at home after ambulatory hand surgery has also been analysed [30]. However, the analgesic efficacy of a combination of paracetamol and metamizole for pain relief at home after day surgery has never been studied.

Hence, in the present study we aim to assess if a combination of metamizole and paracetamol is non-inferior to a combination of the NSAID ibuprofen and paracetamol in the treatment of acute postoperative pain at home after painful day case surgery. We hypothesise that ambulatory patients postoperatively treated with paracetamol/metamizole will achieve equal or even better pain relief compared to patients treated with paracetamol/ibuprofen. In addition, we aim to assess and compare QOR profiles of both groups. We hypothesise that each type of day surgery included in our trial will have a unique QOR profile, significantly different from the QOR profile of other types of day surgery.

## Methods

### Study design

This is an investigator-initiated, double-blind, randomised controlled, non-inferiority trial. The study is being performed in accordance with the Declaration of Helsinki and has been approved by the ethics committee of the JESSA Hospital Hasselt (registration number 15.105/pijn15.02) and by the European Union Drug Regulating Authorities Clinical Trials (EudraCT Number 2015-003987-35). Informed consent will be obtained from all participants. The final report will follow the CONSORT 2010 guidelines as well as the extension to non-inferiority trials.

### Population

Consecutive adult patients undergoing elective haemorrhoid surgery, arthroscopic shoulder or knee surgery, or inguinal hernia repair in a day care setting will be informed about the study on their preoperative visit by the surgeon and they will be provided with a patient information sheet.

Patients will be assessed for eligibility on arrival at the outpatient clinic. Detailed eligibility criteria are listed in Table 1. The purpose, procedures, and potential risks and benefits of the study will be explained thoroughly to all participants who meet the eligibility criteria by a trained resident physician or study nurse. If interested, a written informed consent will be obtained from each participant prior to randomisation. The participants will be able to withdraw from the study at any time without consequences for therapy. This trial will be performed at a high-volume institution (JESSA Hospital, Hasselt, Belgium). Based on the number of selected procedures performed annually in the JESSA hospital, it is estimated that the trial will be executed from 28 January 2016 to January 2017, including enrollment and follow-up.

**Table 1:** Eligibility criteria

Inclusion criteria	Exclusion criteria
- Patients aged between 18 and 70 years - ASA classification 1, 2 or 3 - Body weight > 50 kg - One of the following ambulatory surgical procedures: • Haemorrhoid surgery • Arthroscopy knee • Arthroscopy shoulder • Inguinal hernia repair	- Not meeting inclusion criteria - Cognitive impairment or no understanding of the Dutch language - Preoperative pharmacologic pain treatment and/or a history of chronic pain - Allergy to or a contraindication for taking the study medication (e.g. paracetamol, metamizole, ibuprofen or another non-steroidal anti-inflammatory drug) - Porphyria - Pregnancy or lactation - A history of severe renal, hepatic, pulmonary, or cardiac failure - Current symptoms or a history of gastrointestinal bleeding - Ileus or chronic obstipation - A history of substance abuse, or use of medication with a suppressive effect on the central nervous system - Hypotension - Hematological disease - Use of anti-rheumatic drugs - Rhinosinusitis or nasal polyposis - Glucose-6-phosphate dehydrogenase deficiency - Fever or other signs of infection - For patients undergoing arthroscopy shoulder: refusal of an interscalene block

### Interventions

After obtaining written informed consent, patients will be randomised in a 1:1 ratio to either of the two study groups: a combination of metamizole and paracetamol (MP) or a combination of ibuprofen and paracetamol (IP) group. Patients in the MP group (experimental arm) will be instructed to take metamizole 1 g orally three times a day for 4 days and patients in the IP group (control arm) will be instructed to take ibuprofen 600 mg orally three times a day for 4 days. All patients will also be treated with paracetamol 1 g orally four times a day during the entire study period. The first dose of study medication (metamizole and paracetamol (MP) or ibuprofen and paracetamol (IP) will be given 30 minutes before surgery. Rescue medication consists of tramadol 50 mg orally and will be taken up to three times a day only if pain relief was not satisfactory with the study medication. Patients will be instructed to take their trial medication as prescribed and will be provided with a detailed medication schedule. Furthermore, they will be called by telephone daily and asked if they took their trial medication as prescribed.

### Perioperative procedure

All patients scheduled for an arthroscopic shoulder procedure will receive an interscalene block preoperatively.

In addition, general anaesthesia will be induced with intravenous (IV) alfentanil 10 mcg/kg IV sufentanil 0.15 mcg/kg and IV propofol 2 mg/kg. Patients undergoing arthroscopic shoulder surgery or laparoscopic inguinal hernia repair will also receive rocuronium 20–40 mg before endotracheal intubation. A laryngeal mask airway will be inserted in all other patients. Anaesthesia will be maintained with sevoflurane in a mixture of 50:50 air/oxygen. Before the end of surgery, all patients will receive ondansetron 4 mg IV. Furthermore, wound infiltration with local anaesthesia (bupivacaine 0.5 %) will be performed in all patients except those receiving an interscalene block. Duration of surgery will be recorded.

Postoperatively, all patients will be treated with subsequent bolus injections of piritramide 2 mg intravenously until a Numeric Rating Scale (NRS) ≤3 is reached/achieved in the Post-Anaesthesia Care Unit (PACU). Before hospital discharge, patients will receive the study medication and instructions for use. Dropout criteria are surgical complications leading to either resurgery or unanticipated hospital admission.

### Outcome measures

**Fig. 1:**
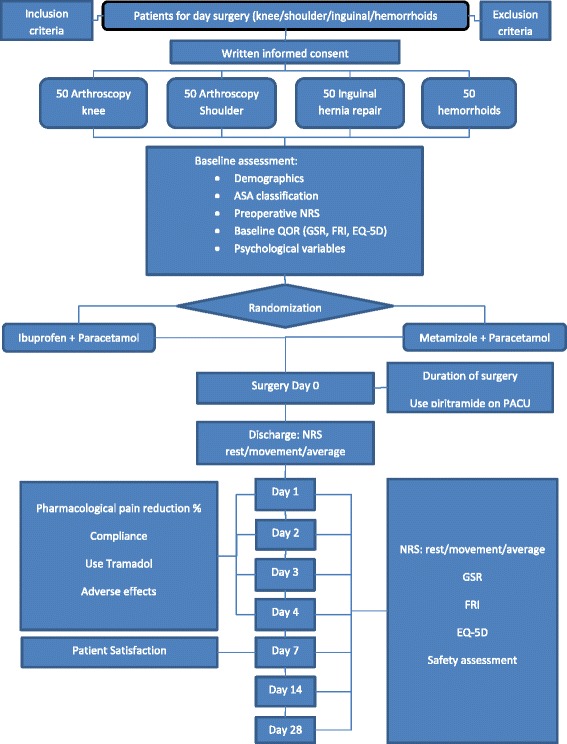
Schedule of enrollment, interventions and assessments

Outcome measures will be assessed at baseline and by telephone call at day 0, 1, 2, 3, 4, 7, 14 and 28 postoperatively. Several attempts at different times will be repeated in case of an initial unsuccessful call attempt. Outcomes in the domains of pain, QOR, compliance with study medication and patient satisfaction will be assessed (Fig. 1).

### Primary outcome measures

Primary endpoints are:

1. Average postoperative pain intensity measured by an 11-point Numeric Rating Scale (NRS; where 0 = no pain, and 10 = worst pain imaginable) at postoperative day 1.
2. QOR profile measured by the Functional Recovery Index (FRI) [1], the 1-item Global Surgical Recovery (GSR) index [31] and the 5-dimensional European Quality of Life (EQ-5D) questionnaire [32] at days 1, 2, 3, 4, 7, 14 and 28 postoperatively.
  The convenient and also validated GSR index represents a single question about the extent to which patients considered themselves to be recovered from the surgery (0–100 %) [31].
  The FRI is a questionnaire-based instrument specifically developed to assess post-discharge functional QOR after day surgery and covers 14 items grouped under three factors (pain and social activity, lower limb activity and general physical activity) [1].
  The 5-dimensional EQ-5D is a non-disease-specific instrument developed for describing and valuing health-related quality of life [32] and has already been used to assess intermediate (4 days) and late (2 weeks to 6 months) quality of recovery after day surgery [3, 33, 34].

### Secondary outcome measures

The following outcomes will also be assessed (see also Fig. 1):

- Postoperative pain intensity at movement and rest measured by an 11-point Numeric Rating Scale (NRS; where 0 = no pain, and 10 = worst pain imaginable) at discharge and day 1, 2, 3, 4, 7, 14 and 28 postoperatively.
- Average postoperative pain intensity measured by an 11-point Numeric Rating Scale (NRS; where 0 = no pain, and 10 = worst pain imaginable) at day 0, 2, 3, 4, 7, 14 and 28 postoperatively.
- Adherence to study medication at day 1, 2, 3 and 4 postoperatively.
  - Definition compliance: full compliance: analgesia use as prescribed “Yes”, no compliance: analgesia use as prescribed “No”.
- Adverse effects of study medication (i.e. pyrosis, signs of agranulocytosis or thrombocytopenia)
- Total postoperative intravenous piritramide consumption at the PACU (milligram)
- Use of rescue medication (tramadol at home) at day 1, 2, 3 and 4 postoperatively (yes/no).
- Satisfaction with study medication, surgery and hospital care and telephone follow-up measured at the seventh postoperative day by an 11-point Numeric Rating Scale (NRS).

### Baseline assessment measurements

In addition to the primary and secondary outcome measures, the researcher will also record participants’: age, gender, body mass index (BMI), ASA classification, work status, highest level of education, fear of the surgical procedure (using an 8-item surgical fear questionnaire) [3, 35], preoperative pain (the baseline NRS score), expected pain (NRS score), baseline GSR, FRI and EQ-5D, and the history of (related) surgery.

### Safety assessments

All possible adverse effects (AEs) of study medication will be explained thoroughly to all patients who meet the eligibility criteria. All participants will be questioned about adverse events at each telephone call (day 1, 2, 3, 4, 7, 14 and 28 postoperatively). Patients will be specifically asked whether they experienced postoperative nausea and vomiting, pyrosis or stomach ache, obstipation, anaphylaxis, fever, chills, mouth ulcers, a sore throat or signs of infection, petechiae and bleeding diathesis. Furthermore, patients are instructed to contact a research assistant immediately by phone if they experience moderate to severe signs of infection and/or bleeding diathesis. A complete blood count will then be performed to exclude leukopenia, anaemia and thrombocytopenia and trial medication will be withdrawn from the patient. Such an event will be reported as a serious adverse event (SAE).

### Randomisation and blinding

Participants will be randomly assigned to the MP or the IP group using a computer-generated random allocation sequence, created by the study statistician. Randomisation will be stratified for type of surgery. Each patient will receive a unique randomised test number corresponding to the specified drug, according to the group allocation. The randomisation list remains with the study statistician and the hospital pharmacy for the whole duration of the study. Hence, the patients participating in the trial, the treating physicians, the researchers dispensing the medication and assessing outcomes (i.e. four trained resident physicians and one study nurse) and the data managers will be blinded for group allocation. For test drug blinding, the metamizole tablets and the ibuprofen tablets will be made to be visually lookalike. Furthermore, the hospital pharmacy will package study medication into identical blister containers. Each container will be numbered according to the randomisation list. Only adverse events (AEs) that are considered serious, unexpected and at least possibly related to the medication would have to be unblinded.

### Sample size

The primary outcome measures are the average postoperative pain intensity measured by an 11-point Numeric Rating Scale (NRS) on the first postoperative day as well as the QOR profile. As QOR profile is strongly influenced by postoperative pain intensity, sample size calculation will be based on expected postoperative pain intensity. Based on previous studies, we assume a standard deviation of the NRS scores of 2.5 on the first postoperative day and 1.7 on the fourth postoperative day [3, 5]. A difference in mean average NRS score of 1 point or less is considered non-inferior. Therefore, the present study must have the power to detect a difference of more than 1 point to reject the null hypothesis that the analgesic power of a combination of metamizole and paracetamol is inferior compared to a combination of ibuprofen and paracetamol. Based on these assumptions, we will require 78 patients in each group to have a power of at least 80 % (β = 0.2). To determine non-inferiority, we will compute 95 % confidence intervals for the difference in primary endpoints. The sample size will be inflated to 100 participants per group (200 in total) to account for a possible 22 % loss to follow-up.

### Data management and statistical analysis

Participants’ data will be recorded in individual participant record booklets. Coded, depersonalised data will be entered into a web-based questionnaire (Questback) and then exported to SPSS version 21 (IBM Corp, Armonk, NY, USA). Database access will be restricted to the authorised research team. Participants’ study information will not be released outside of the study without the written permission of the participant.

All primary and secondary endpoints will be analysed on a per protocol basis according to a non-inferiority design. As a sensitivity analysis, we will compare these results to an intention-to-treat analysis. Data will be presented as mean values +/- SD, numbers (n), and percentages (%). *P* values < 0.05 will be considered statistically significant. Missing baseline values will be imputed using multiple imputation. The number of imputations will be set to 10. To determine non-inferiority for the difference in NRS score on the first day after surgery, we will compute 95 % confidence intervals. The QOR profiles will be assessed using linear mixed models, taking time, type of surgery and group assignment into account. Differences between the groups on secondary outcomes will be analysed using the Student’s *t* test for continuous outcomes, except for the pain scores, since these are often heavily skewed. For those outcomes, we will use the Mann-Whitney *U* test. All categorical variables will be compared using Pearson’s χ2 test. For multivariate analysis the following potential confounders will be assessed: surgical fear, preoperative pain (baseline NRS), expected pain, baseline FRI and EQ-5D, age, sex, work status, educational level, ASA classification, BMI, history of related surgery, type of surgery, and duration of surgery. In addition, changes in the QOR scores over time in the postoperative period will be analysed using linear mixed models and we will use logistic regression to explore possible predictors for QOR. All analyses will be performed using SPSS version 21.

### Monitoring

#### Data Monitoring Committee (DMC)

The major task of the DMC is safety monitoring. Any SAE or suspected unexpected serious adverse reaction liable to produce disability or deformity is an indication to set up a DMC meeting. Members of the DMC are (1) head of the Department of Hematology of the JESSA Hospital Hasselt and (2) the primary statistician of the trial. The advice of the DMC will be notified by the research committee to the ethics committee of the JESSA Hospital Hasselt that approved the protocol. Interim analyses will not be performed by the DMC to mitigate possible bias and preserve trial integrity.

### Procedures for recording and reporting adverse events

Adverse events (AEs) will be recorded and severity and relation to study participation will be assessed. SAE data will be collected and handled by the DMC.

## Discussion

This study will provide clinical evidence on the analgesic efficacy and safety of a combination of metamizole and paracetamol in treating postoperative pain at home after painful day surgery.

Metamizole was first marketed in Germany in 1922 [36]. The molecular mechanism of analgesic and antipyretic action of metamizole however, is still not fully understood. Metamizole is a prodrug and its activity is due to its immediate conversion to its active metabolites [37]. Several mechanisms were proposed, including the involvement of endogenous opioids and COX-1/COX-2 inhibition by dipyrone and its metabolites [38, 39]. Recently, there is growing evidence that also the endocannabinoid/endovanilloid system plays a role in the effects of metamizole against pain [36, 37, 40, 41]. It has been additionally emphasised that co-administration of tramadol and dipyrone results in an important potentiation of their individual anti-nociceptive effects [42, 43]. Another important advantage of metamizole is the favourable gastrointestinal [44, 45], cardiovascular and cerebrovascular profile compared to NSAIDs [16–20]. Consequently, the excess mortality due to agranulocytosis, aplastic anaemia, anaphylaxis and serious gastrointestinal complications was found to be in favour of metamizole compared to the NSAID diclofenac (25 per 100 million vs 592 per 100 million) [46].

In contrast, use of metamizole is under debate and is even restricted in many countries due its association with agranulocytosis. This restriction is based on two studies who found a very high incidence of metamizole-induced agranulocytosis (MIA) [47, 48]. However, recent literature reported a very limited incidence of approximately 0.5 to 1 MIA case per million per year [22, 49–52]. From this it is concluded that the absolute risk of serious adverse effects (including MIA) after postoperative treatment with metamizole is probably much lower than the absolute risk of serious adverse effects after treatment with NSAIDs.

This study also aims to provide data on QOR at different time points after four types of day surgery, each known to have a high incidence of poor QOR at the fourth postoperative day [3].

There are several instruments for assessing the intermediate (4 to 7 days) and late (1 month) QOR after day surgery, including the Functional Recovery Index (FRI) and Global Surgery Recovery (GSR) index [1, 2, 31]. Also, quality of life questionnaires may fulfill the requirements as useful indicators of surgical recovery [4]. However, so far there is no general agreement on the optimal instruments for evaluating recovery and outcome following ambulatory surgery [53]. In our opinion, the FRI is the best instrument for evaluating QOR at home after day surgery. In our study we will apply three different instruments to assess QOR: the Global Surgical Recovery index (GSR), the FRI and the quality of life questionnaire EQ-5D. QOR will be assessed at days 1, 2, 3, 4, 7, 14 and 28 postoperatively.

In conclusion, this trial aims to test the non-inferiority of the analgesic power of a combination of metamizole and paracetamol compared to a combination of ibuprofen and paracetamol after painful day surgery. This study may also provide an insight into the QOR profile after four different types of surgery and into the interrelationship between the three different instruments used to assess QOR.

## Abbreviations

AE: Adverse effect
BMI: Body mass index
DMC: Data Monitoring Committee
EQ-5D: 5-dimensional European Quality of Life questionnaire
FRI: Functional Recovery Index
GSR: Global Surgical Recovery
IP: Ibuprofen and paracetamol
IV: Intravenous
MIA: Metamizole-induced agranulocytosis
MP: Metamizole and paracetamol
NRS: Numeric Rating Scale
NSAID: Non-steroidal anti-inflammatory drug
PACU: Post-Anaesthesia Care Unit
QOR: Quality of recovery
SAE: Serious adverse reaction
